# Identification of leaves of wild Ussurian Pear (*Pyrus ussuriensis*) based on YOLOv10n-MCS

**DOI:** 10.3389/fpls.2025.1588626

**Published:** 2025-07-03

**Authors:** Niman Li, Xingguang Dong, Yongqing Wu, Luming Tian, Ying Zhang, Hongliang Huo, Dan Qi, Jiayu Xu, Chao Liu, Zhiyan Chen, Yulu Mou

**Affiliations:** ^1^ Research Institute of Pomology, Chinese Academy of Agricultural Sciences (CAAS), Key Laboratory of Horticulture Crops Germplasm Resources Utilization, Ministry of Agriculture and Rural Affairs, Xingcheng, China; ^2^ School of Software, Liaoning Technical University, Huludao, China

**Keywords:** identification, Ussurian Pear, leaves, YOLOv10n, target detection

## Abstract

**Introduction:**

Wild Ussurian Pear germplasm resource has rich genetic diversity, which is the basis for genetic improvement of pear varieties. Accurately and efficiently identifying wild Ussurian Pear accession is a prerequisite for germplasm conservation and utilization.

**Methods:**

We proposed YOLOv10n-MCS, an improved model featuring: (1) Mixed Local Channel Attention (MLCA) module for enhanced feature extraction, (2) Simplified Spatial Pyramid Pooling-Fast (SimSPPF) for multi-scale feature capture, and (3) C2f_SCConv backbone to reduce computational redundancy. The model was trained on a self-made dataset of 16,079 wild Ussurian Pear leaves images.

**Results:**

Experiment results demonstrate that the precision, recall, mAP50, parameters, FLOPs, and model size of YOLOv10n-MCS reached 97.7(95% CI: 97.18 to 98.16)%, 93.5(95% CI: 92.57 to 94.36)%, 98.8(95% CI: 98.57 to 99.03)%, 2.52M, 8.2G, and 5.4MB, respectively. The precision, recall, and mAP50 are significant improved of 2.9%, 2.3%, and 1.5% respectively over the YOLOv10n model (p<0.05). Comparative experiments confirmed its advantages in precision, model complexity, model size, and other aspects.

**Discussion:**

This lightweight model enables real-time wild Ussurian Pear identification in natural environments, providing technical support for germplasm conservation and crop variety identification.

## 1 Introduction

The genus Pear is a worldwide fruit tree, which originated in the mountainous areas of southwest China in the Tertiary Period, and evolved into two groups of Occidental and Oriental pear, respectively and there are 22 primary *Pyrus* species recognized by the academic community (Bell, 1996). China is the center of origin of oriental pear, with 13 native species, including 5 basic species: *P. pyrifolia*, *P. ussuriensis*, *P. pashia*, *P. betulaefolia*, and *P. calleryana* (Pu and Wang, 1963). Among them, the Ussurian Pear (*P. ussuriensis*) is the most cold-tolerant species, with wild and cultivated types, which grow in northeast China, northern Hebei and northern Shaanxi, and also have wild distribution in the Russian Far East and North Korea. Northeast China is the characteristic production area of Ussurian Pear, and there are more than 150 cultivated varieties. The fruit of Ussurian Pear is generally small, round, rich in flavor, and eaten after ripening and softening, The representative varieties are Nanguo, Jingbai, Anli and so on, which are deeply loved by the local people. It is generally thought that the cultivars have been domesticated from wild species, and the diversity of wild species is more abundant, and our study of the genetic diversity of the Ussurian Pear has reached the same conclusion (Cao et al., 2012). The wild Ussurian Pear are mainly used as rootstocks in Northeast China, and their ecological and production value are far from being explored. Due to its long-term wild state, wild Ussurian Pear has strong adaptability to the environment, and the diversity of biological and botanical characteristics is also very rich, and these excellent traits are important gene sources for cultivated species improvement (Thakur et al., 2024).

Accurate and rapid identification of germplasm resources is the foundation for their preservation, research, and utilization. However, due to interspecific and intraspecific hybridization being the main mode of pear evolution, pear populations are large and with a high degree of heterozygosity. This leads to significant difficulties in identifying and classifying pear germplasm resources (Xue et al., 2017). Morphological identification is the most fundamental and important method for pear variety identification (Zhang et al., 2022a). The staff identifies pear varieties by analyzing their phenotypic characteristics, such as observing leaf morphology, branch color, and fruit characteristics. However, due to the susceptibility of this method to individual plant development, environmental conditions, cultivation measures, and human factors, the identification process is time-consuming and has a high error rate (Adão et al., 2025). With the rapid development of high-throughput sequencing and molecular biology techniques, pear varieties can be accurately identified using molecular markers. However, this method has limitations such as cumbersome operation, time-consuming, and high cost due to the high requirements for operating steps and equipment (Liu et al., 2024).

In the study of plant variety identification, leaves are easier to collect than other organs. The leaves of any plant have their unique characteristics, such as leaf shape, leaf bases, leaf apex, and leaf margin (Wang et al., 2023b). Pear leaves are mainly divided into circular, ovate, elliptical, lanceolate, and lobed shapes. Among them, ovate shaped resources are the most abundant. Leaf base refers to the proximal part of the leaf near the stem. The shapes of pear leaf bases are mainly divided into narrow cuneate, cuneate, broad cuneate, round, truncate, and cordate, with broad cuneate being the most prevalent. The shapes of pear leaf apices can be divided into acuminate, obtuse, acute, and caudate. And the leaf margins can be classified as entire, crenate, obtusely serrate, sharply serrate, and doubly serrate. In recent years, with the advancement of agricultural informatization, computer vision, machine learning and other technologies are widely used different areas in agriculture (Zhang et al., 2022b). More and more researchers are focusing on image classification and pattern recognition, and using them to quickly identify and classify plant leaves. Pan et al. (2024) used four deep learning networks, including GoogleNet, ResNet50, ResNet101, and VGG16, to identify and classify the leaf images of 23 wild grapes, and realized automatic real-time identification of wild grapes. Chen et al. (2022) used Convolutional Neural Network (CNN) to extract features and classify 30 types of apple leaves, significantly improving the classification accuracy of apple cultivars. Wei Tan et al. (2018) proposed a CNN model called the D-leaf model, which utilizes leaf vein morphometric to classify plant species. And three different CNN models (pre trained AlexNet, fine-tuned AlexNet, and D-Leaf) were used to preprocess the leaf images and extract feature information. To classify the extracted features, five machine learning approaches were utilized, including Artificial Neural Networks (ANN), Support Vector Machines (SVM), and CNN, etc. The results confirm that deep learning-based models are effective for tasks such as leaf recognition and variety categorization.

Although the use of deep learning for leaf classification is highly effective, many of the models used have limitations such as complex network structures and large parameter quantities, which consume a significant amount of computing resources and can only be deployed on the server side (Fang et al., 2019), resulting in certain limitations in leaf recognition tasks.”You Only Look Once” (YOLO) target detection algorithm is the leading single-stage algorithm in deep learning object detection methods (Adarsh et al., 2020). It can predict the category and location of objects in the image by recognizing image information. In recent years, with the continuous innovation of the YOLO series (Redmon, 2016; Redmon and Farhadi, 2017; Farhadi and Redmon, 2018; Bochkovskiy et al., 2020; Jocher, 2020; Li et al., 2022; Wang et al., 2023a; Jocher et al., 2023; Wang et al., 2025a, 2024), its performance has gradually improved, achieving a balance between recognition speed and accuracy. It has better performance and application prospects in quickly identifying plant leaves and deploying on mobile devices. The leaves of different pear varieties have differences in shape, leaf apex, and leaf margin. These differences can serve as the basis for YOLO classification. And the characteristics of pear leaves are highly matched with YOLO’s advantages such as real-time detection and multi-target processing. YOLO can extract these morphological features through CNN and learn the differentiation patterns of different varieties through training. In addition, by optimizing the model, YOLO’s potential for using leaves to classify pear varieties can be fully utilized. Many recent studies have demonstrated that YOLO series target detection algorithms can effectively achieve leaf species identification and classification tasks. Yang et al. (2024) used the YOLOX algorithm and combined with the self-made tea bud dataset to establish a tea bud classification model, which can recognize and classify four types of tea buds, in which the recognition accuracy for the yellow mountain species could reach 90.54%. Niu et al. (2024) improved the YOLOv8 algorithm and proposed a lightweight YOLOv8-EFS model based on their own soybean seedlings and weeds image datasets, which can quickly and accurately identify multiple weed species and provide support for intelligent weed control in farmland management and unmanned farms.

The rapid and accurate identification of wild pear accessions relies on high-quality datasets and high-performance detection models. Although significant achievements have been made in plant species recognition through existing research, there are still problems such as excessive consumption of computational resources and low recognition accuracy in the models used for recognition. In addition, there is currently limited research on the identification of wild pear germplasm resource, and there have been no reports on the use of target detection algorithms to identify wild pear leaves for variety classification.

In response to the above issues, this article takes wild Ussurian Pear leaves in natural environments as the research object, selects YOLOv10n as the baseline model, and proposes an improved leaves recognition and classification model YOLOv10n-MCS. Firstly, this study selected 30 wild Ussurian Pear accessions and constructed a dataset of wild Ussurian Pear leaves images. Collect 500–600 images for each accession to meet the requirements of multitasking classification. Secondly, based on YOLOv10n, Mixed Local Channel Attention (MLCA) module, C2f SCConv module, and Simplified Spatial Pyramid Pooling - Fast (SimSPPF) module were introduced to enhance the feature extraction ability of the model, reduced computational redundancy, and improve detection performance. Finally, the improved model was combined with the dataset of wild Ussurian Pear leaves images to establish a recognition and classification model covering 30 wild Ussurian Pear accessions, thereby achieving automatic recognition and classification of wild Ussurian Pear leaves. This study can accurately and quickly identify and classify wild Ussurian Pear leaves, reducing complicated labor costs. In addition, it also provides reference for the protection, utilization, classification research of wild pear germplasm resource, as well as the identification of other crop varieties.

## 2 Materials and methods

### 2.1 Image data acquisition

**Figure 1:**
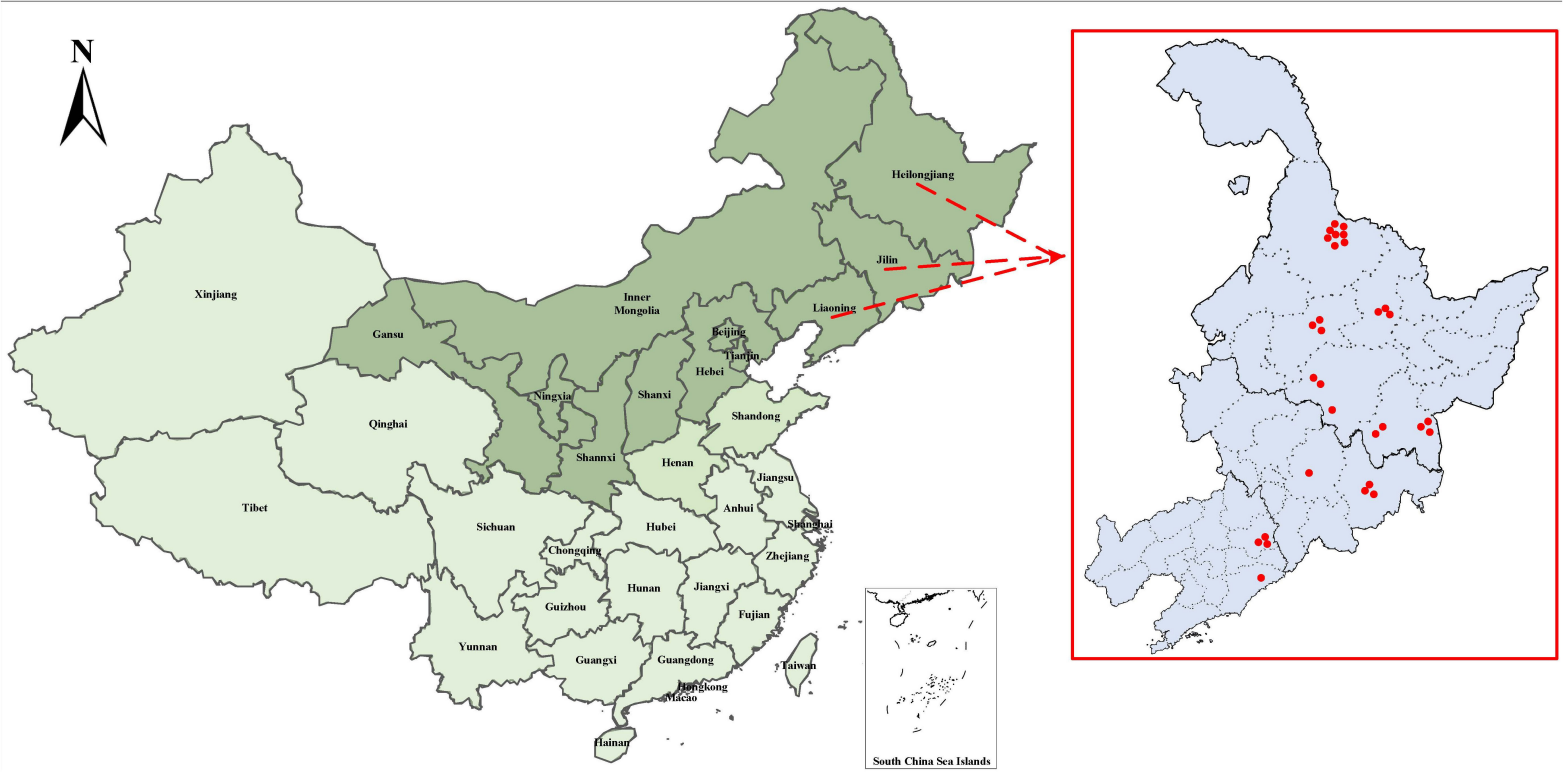
Map of the origin regions of wild Ussurian Pear accession.

**Figure 2:**
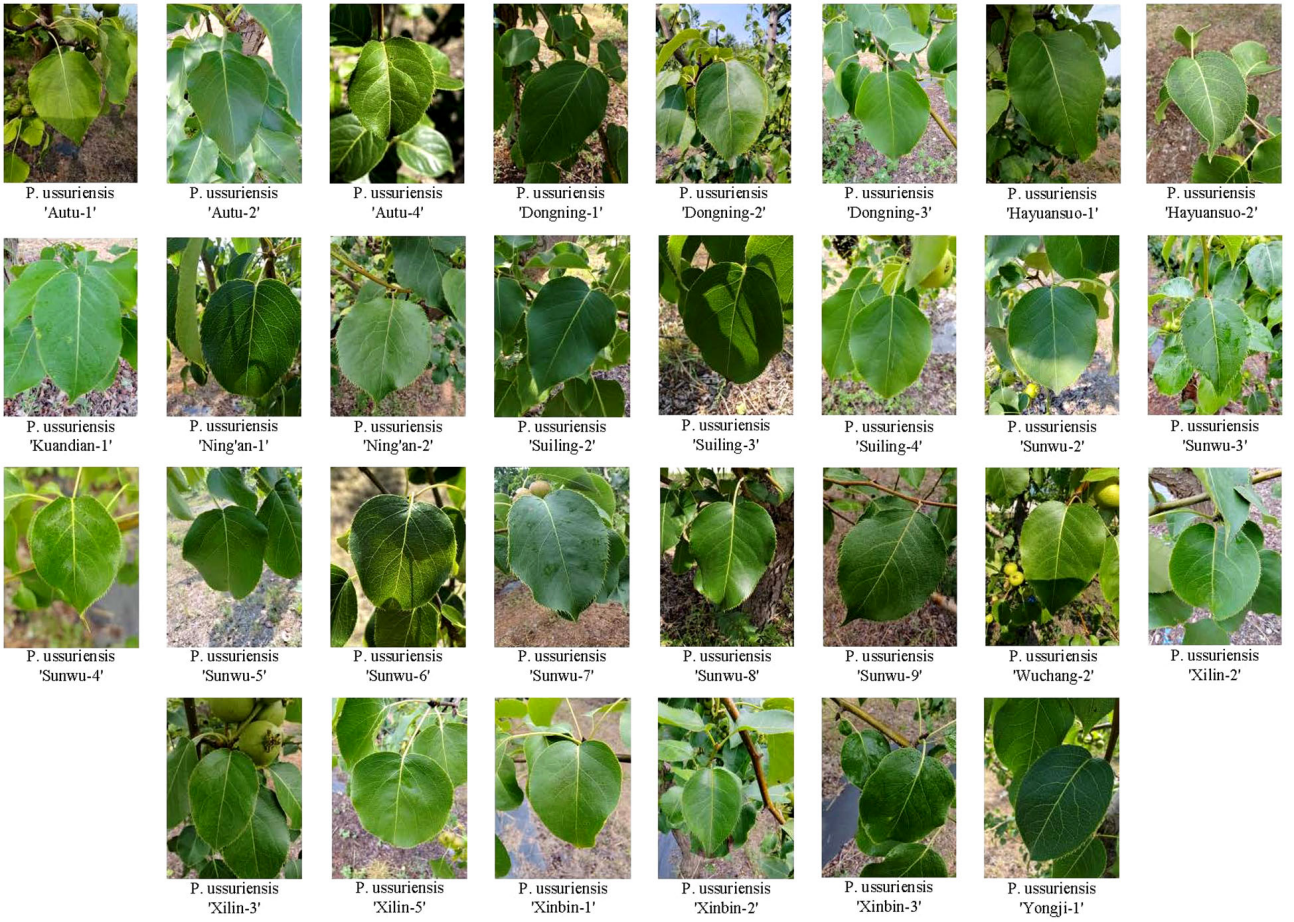
Some representative samples of the leaves images dataset.

Training a wild pear leaves recognition and classification model requires inputting a large number of pear leaves images as sample images into the network model. Model accuracy and efficiency are significantly influenced by the quality of the dataset used for training. We selected 30 wild Ussurian Pear accessions as the research objects for wild pear leaves recognition and classification, and constructed a natural background wild Ussurian Pear leaves images dataset. The origin of these 30 wild Ussurian Pear accessions is shown in **Figure 1**. We went to the “National Pear and Apple Germplasm Resources Repository (Xingcheng)” to collect images of wild Ussurian Pear leaves. From October 5th to 12th, 2024, use a high-resolution camera on the same mobile phone to capture images of leaves from 30 wild pear resources under natural conditions. Place the camera at a distance of 20-60cm from the leaves, take frontal images of adult leaves, and try to ensure that the leaf shape, leaf bases, leaf apex, and leaf margin in the image are clear as much as possible. Collect 500–600 images for each variety. The collected images are in JPG format with a resolution of 3072 pixels × 4096 pixels. The weather conditions during the shooting process include sunny, cloudy, and rainy days. There are both shooting under soft lighting and strong afternoon lighting, covering various common weather conditions and natural lighting conditions, increasing the diversity and complexity of the natural conditions in which the leaves are located. Ultimately, a total of 16997 images of wild Ussurian Pear leaves were obtained through this process, and some of the leaves images dataset samples are shown in **Figure 2**.

### 2.2 Images annotation and dataset construction

After careful manual screening, images with clear leaves contours and textures were selected, and a total of 16079 leaves images were used to form the wild Ussurian Pear leaves images dataset. Manually annotate the filtered leaves images using LabelImg software(can be in https://github.com/HumanSignal/labelImgget). Use the “Create RectBox” function in the software to annotate each leaf based on the minimum external rectangle in the image, and select a category for the annotated blade, such as’Sunwu-3’. In order to avoid inaccurate labeling and blurry targets affecting the quality and performance of the model, blades with an occlusion area exceeding 70% are not labeled. And ensure that the rectangular box contains as little background as possible to improve recognition precision. The annotation example is shown in Image 1 of the **Supplementary File**. Subsequently, after annotation, obtain the.txt format annotation file required by YOLO. This file contains the category of the blade, the coordinates x and y of the center point of the rectangle, the width w and height h relative to the image Fan et al. (2024). To facilitate model training and evaluation, the wild Ussurian Pear leaves images dataset was randomly split into training, validation, and test sets in a 7:2:1 ratio, corresponding to model training, validation, and prediction tasks, respectively. The training set contained 11243 samples, the validation set included 3371 samples, and the test set comprised 1465 samples. Detailed sample information is provided in **Table 1**.

**Table 1:** Leaves samples information of 30 species of wild pear.

No.	Name	Origin	Trained	
			Number of images	Number of labels
0	*P. ussuriensis* ‘Wuchang-2’	Heilongjiang Wuchang	361	508
1	*P. ussuriensis* ‘Sunwu-8’	Heilongjiang Sunwu	354	498
2	*P. ussuriensis* ‘Sunwu-6’	Heilongjiang Sunwu	378	475
3	*P. ussuriensis* ‘Ning’an-1’	Heilongjiang Ning’an	368	480
4	*P. ussuriensis* ‘Autu-4’	Jilin Antu	404	497
5	*P. ussuriensis* ‘Dongning-3’	Heilongjiang Dongning	372	487
6	*P. ussuriensis* ‘Xilin-3’	Heilongjiang Xilin	361	490
7	*P. ussuriensis* ‘Ning’an-2’	Heilongjiang Ning’an	403	506
8	*P. ussuriensis* ‘Xilin-2’	Heilongjiang Xilin	375	526
9	*P. ussuriensis* ‘Sunwu-3’	Heilongjiang Sunwu	365	530
10	*P. ussuriensis* ‘Sunwu-7’	Heilongjiang Sunwu	363	530
11	*P. ussuriensis* ‘Dongning-1’	Heilongjiang Dongning	413	512
12	*P. ussuriensis* ‘Xilin-5’	Heilongjiang Xilin	375	498
13	*P. ussuriensis* ‘Dongning-2’	Heilongjiang Dongning	364	529
14	*P. ussuriensis* ‘Sunwu-2’	Heilongjiang Sunwu	385	510
15	*P. ussuriensis* ‘Suiling-3’	Heilongjiang Suiling	365	501
16	*P. ussuriensis* ‘Suiling-2’	Heilongjiang Suiling	362	478
17	*P. ussuriensis* ‘Xinbin-1’	Liaoning Xinbin	391	517
18	*P. ussuriensis* ‘Hayuansuo-1’	Heilongjiang Haerbin	357	502
19	*P. ussuriensis* ‘Autu-2’	Jilin Antu	391	509
20	*P. ussuriensis* ‘Yongji-1’	Jilin Yongji	376	510
21	*P. ussuriensis* ‘Xinbin-3’	Liaoning Xinbin	389	490
22	*P. ussuriensis* ‘Kuandian-1’	Liaoning Kuandian	371	507
23	*P. ussuriensis* ‘Sunwu-4’	Heilongjiang Sunwu	381	482
24	*P. ussuriensis* ‘Sunwu-9’	Heilongjiang Sunwu	366	468
25	*P. ussuriensis* ‘Hayuansuo-2’	Heilongjiang Haerbin	364	505
26	*P. ussuriensis* ‘Xinbin-2’	Liaoning Xinbin	364	481
27	*P. ussuriensis* ‘Suiling-4’	Heilongjiang Suiling	387	507
28	*P. ussuriensis* ‘Autu-1’	Jilin Antu	368	501
29	*P. ussuriensis* ‘Sunwu-5’	Heilongjiang Sunwu	370	482

### 2.3 YOLOv10n model

YOLO is a single-stage target detection algorithm. From focusing on detection speed in YOLOv1 to optimizing the overall architecture, balancing speed, accuracy, and model size in YOLOv10, each version update is gradually addressing the shortcomings of the previous YOLO series. Among them, the YOLOv10 algorithm was proposed by researchers Wang et al. from Tsinghua University in 2024. It addresses the shortcomings in terms of post-processing and model architecture of previous versions of YOLO. Compared to previous versions, it improves both detection precision and speed.

The YOLOv10 network architecture consists of three parts: backbone, neck, and head. It selects YOLOv8 as the baseline model and proposes a new model design based on it. It introduces compact inverted block (CIB) and designs a C2fCIB structure to reduce computational costs and improve efficiency. YOLOv10 proposes an efficient partial self-attention (PSA) module design that improves detection performance and efficiency without increasing excessive computational costs. The head section employs a dual label assignment strategy, which combines a one-to-many approach with a one-to-one matching mechanism. This approach removes the need for non-maximum suppression (NMS), significantly reducing inference latency.

YOLOv10 performs well on COCO Lin et al. (2014), maintaining high accuracy in complex background detection while also being lightweight and easy to deploy on embedded devices. The YOLOv10 includes six variants, namely YOLOv10n, YOLOv10s, YOLOv10m, YOLOv10b, YOLOv10l, and YOLOv10x, to meet different application scenarios. Among them, YOLOv10n is the lightest in terms of parameters and floating-point operations (FLOPs), meeting the lightweight requirements.

### 2.4 Principle of YOLOv10n-MCS model

**Figure 3:**
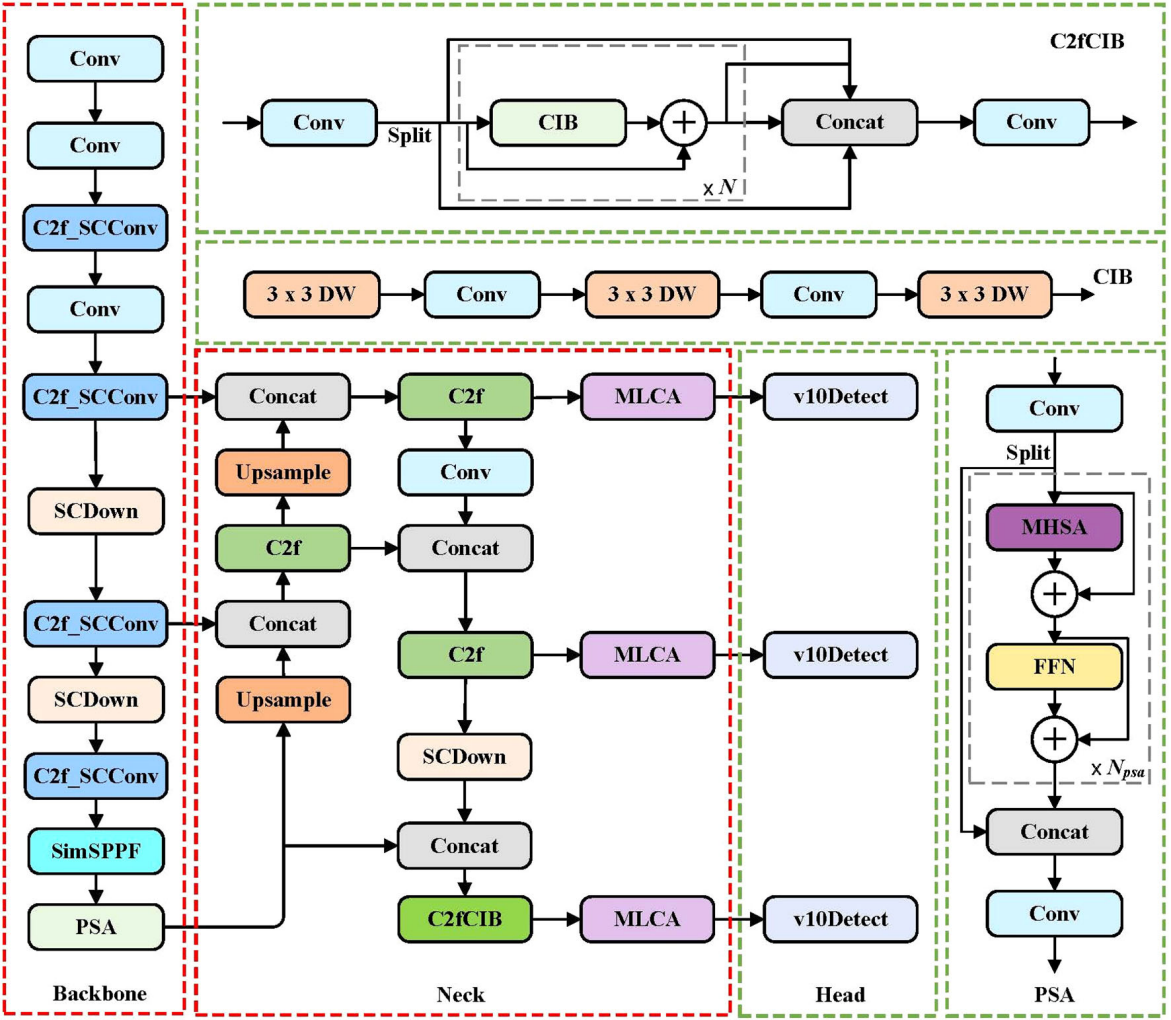
The architecture of the YOLOv10n-MCS model.

In target detection tasks based on deep learning, the size and complexity of the model directly affect the effectiveness of practical applications. Although YOLOv10 algorithm has high recognition accuracy and speed, for wild Ussurian Pear leaves targets in complex scenes, the model still has problems such as slow recognition speed and low precision due to the high similarity of leaf features and occlusion between leaves. Due to the fact that leaves recognition is an efficient and lightweight task, in order to ensure the feature extraction capability of the model for wild Ussurian Pear leaves and enhance the detection performance of the model, this paper selects YOLOv10n as the baseline model and proposes a new YOLOv10n-MCS model. Firstly, the MLCA module is introduced into the neck of the network to enhance the model’s feature extraction capability and improve recognition accuracy. Secondly, using SimSPPF module instead of the original network pyramid pooling layer can improve the detection efficiency of the model. Finally, design a C2f SCConv module to replace C2f in backbone, reducing computational redundancy and improving detection performance. The network structure of the improved YOLOv10n-MCS is shown in **Figure 3**.

#### 2.4.1 The MLCA module

In deep learning, attention mechanisms are designed to emulate the human visual and cognitive processes. By enabling models to concentrate on critical regions while disregarding irrelevant data, attention mechanisms enhance both efficiency and accuracy in information processing. Currently, most channel attention mechanisms, including Squeeze-and-Excitation (SE) Hu et al. (2018) and Efficient Channel Attention (ECA) Wang et al. (2020), focus solely on channel features and neglect spatial information within each channel. This limitation may result in the loss of crucial feature information, negatively impacting model performance in object detection and leading to inaccurate category predictions. Additionally, spatial attention modules, while effective, tend to be computationally intensive. To address this issue, Wan et al. Wan et al. (2023) developed the lightweight MLCA module, which balances performance and complexity. The MLCA module integrates channel and spatial information, along with local and global features, thereby preventing the loss of critical information and enhancing the expressive power and detection performance of object detection algorithms. A better balance has been achieved between detection accuracy, speed, and model parameters without significantly increasing computational costs. Therefore, we incorporated the MLCA module into the neck of our model to improve its feature extraction capabilities.

**Figure 4:**
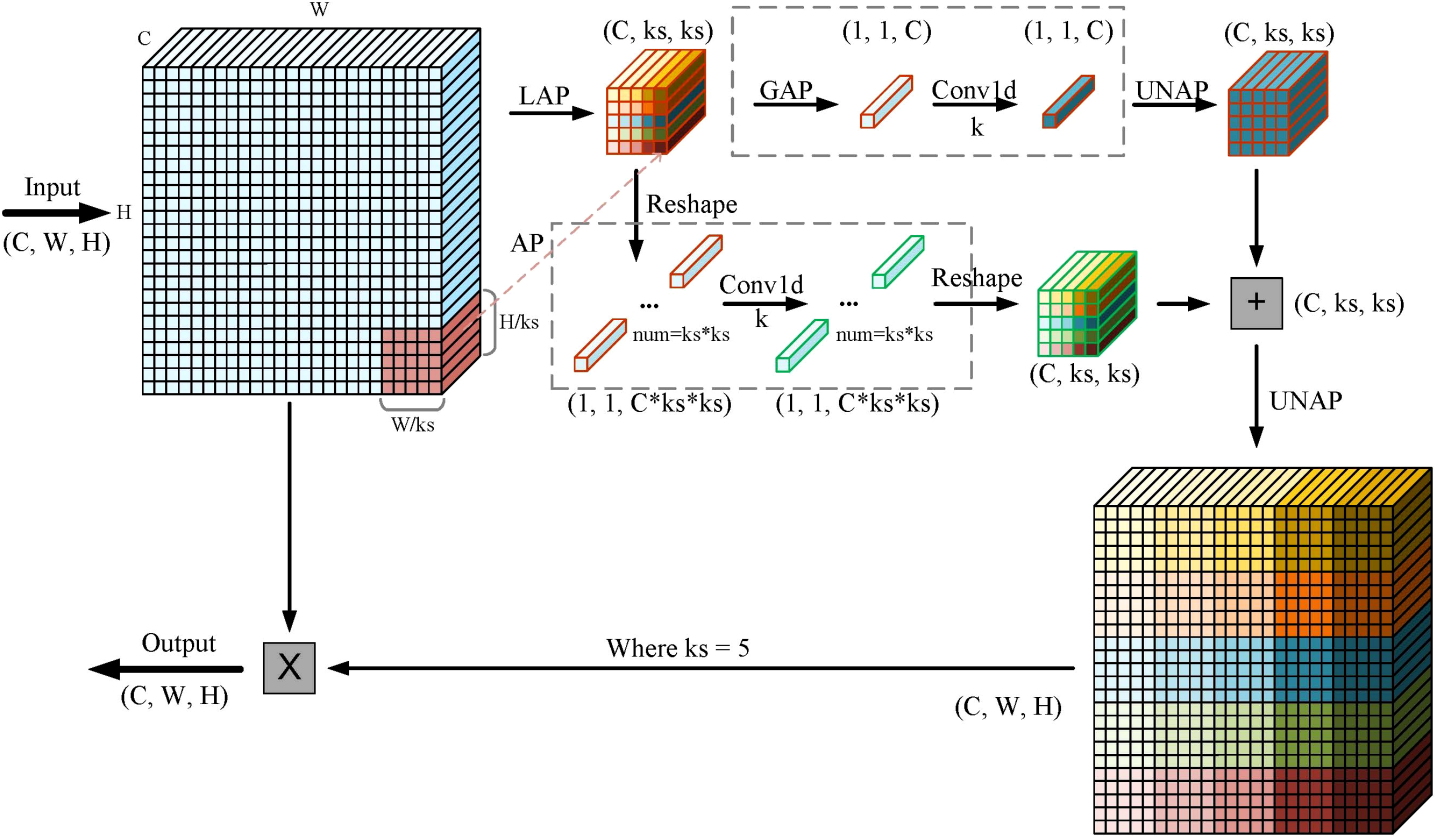
The principle of MLCA algorithm: LAP (Local Average Pooling) which divides the feature map into k * k patches and applies average pooling to each patch; GAP (Global Average Pooling), which uses adaptive pooling to reduce the feature map to a 1 * 1 output size; UNAP (Anti-average Pooling), which mainly focuses on the figure’s properties and scaling to the needed size.


As illustrated in **Figure 4**, the feature vectors are fed into the MLCA module and converted into a 1 * C * ks * ks vector to capture local spatial details. The input is then processed through two parallel branches, each converting the data into one-dimensional vectors. The first branch focuses on local spatial features, while the second branch captures global information. One-dimensional convolution is applied independently to the vectors in both branches. The original resolution of the vectors is recovered through anti-pooling, followed by information fusion to achieve mixed attention. In the figure, k denotes the convolution kernel size, and C represents the channel dimension, with both being proportional. This indicates that local cross-channel interactions are captured by considering only the relationships between each channel and its k neighboring channels. The formula for calculating k is provided in Equation 1. Among them, *γ* and *b* are hyperparameters, odd means that *k* is only odd, and if *k* is even, add 1.

(1) $$k=\Phi \left(C\right)={\left|\frac{{\text{log}}_{2}C)}{\gamma }+\frac{b}{\gamma }\right|}_{odd}$$

#### 2.4.2 The C2f SCConv module

Building a deep network with few parameters and low computational complexity requires compact and highly efficient model design. The C2f module of YOLOv10 enhances its feature extraction capability through the bottleneck structure, but it introduces a large amount of irrelevant interference information for equivalent processing of all channel and position information. The C2f module is internally stacked with a large number of convolution operations, resulting in highly similar features between adjacent channels, leading to redundant features during iteration.

**Figure 5:**
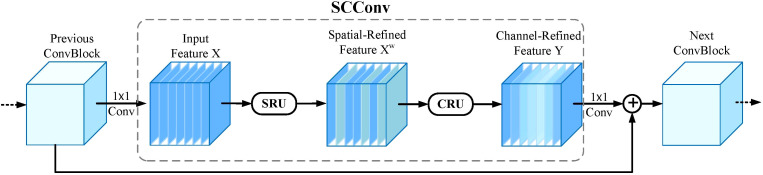
The architecture of SCConv integrated with SRU and CRU.

Spatial and Channel reconstruction Convolution (SCConv), introduced by Li et al. (2023), is an advanced convolutional module designed to minimize spatial and channel redundancy in feature maps, leading to more compact CNN models and enhanced performance. As illustrated in **Figure 5**, the SCConv module comprises two key components: the Spatial Reconstruction Unit (SRU) and the Channel Reconstruction Unit (CRU). The SRU employs a *Separate*-and-*Reconstruct* approach to effectively reduce spatial redundancy. For an input feature *X*, the SRU first performs a *separate* operation, dividing the feature maps into two categories based on their spatial information content: one category includes feature maps with rich spatial details, while the other contains maps with minimal or redundant information. These are then reconstructed to produce the spatially refined feature *X^w^*. The CRU utilizes a *Split-Transform*-and*Fuse* strategy to mitigate channel redundancy. It processes *X^w^* by splitting its channels into two branches. The upper branch employs GWC and PWC convolutions to efficiently extract representative features, while the lower branch uses PWC convolutions to enhance hidden details. To adaptively fuse the features from both branches, the SKNet Li et al. (2019) technique is applied, producing a channel-refined output *Y* that significantly reduces redundancy, computational overhead, and memory usage. Together, the SRU and CRU synergistically reduce redundant information in convolutional layers, lower model complexity, and enhance feature extraction capabilities.

**Figure 6:**
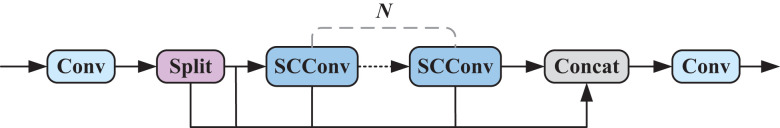
C2f-SCConv module structure diagram.

Therefore, a new C2f SCConv module is designed by replacing the ordinary convolutions in C2f with SCConv. Replace C2f in YOLOv10n’s backbone with C2f SCConv module to improve the model’s learning ability and further reduce the number of model parameters. Reduce redundant features, decrease computational complexity, and improve detection performance. The C2f SCConv module is shown in **Figure 6**.

#### 2.4.3 SimSPPF

Spatial Pyramid Pooling-Fast (SPPF) in YOLOv10n is a pooling operation that introduces three-layer max pooling and connects the outputs of each layer to extract multi-scale information from the input feature map. It extracts and aggregates input feature maps from different perspectives, achieving the fusion of local and global features. Improved computational efficiency while maintaining unchanged performance.

To further enhance the feature extraction capability and training speed of the model, this paper replaces SPPF in the backbone network with SimSPPF. The SimSPPF module is a simplified spatial pyramid pooling module proposed in YOLOv6, which can be used for feature extraction in computer vision tasks.

SimSPPF replaces the activation function Sigmoid Linear Unit (SiLU) of the CBS module in SPPF with a simpler Rectified Linear Unit (ReLU) function, eliminating complex operations and effectively avoiding unnecessary information interference, further improving the computational efficiency of the model. This module first performs convolution operation to compress the input feature map, and then sequentially uses three identical 5 × 5 max pooling layers to obtain feature maps with different receptive field information in a concatenated manner. Then, the feature maps from multiple dimensions are concatenated to finally output the fused feature map.

### 2.5 Evaluation of algorithm performance

In the task of identifying and classifying wild pear leaves, the precision, efficiency, and complexity of the model are the most important aspects. To assess the performance of different models in the task of wild pear leaf identification and classification, this study employed precision (P), recall (R), and mean Average Precision (mAP) as evaluation metrics. The definitions of these metrics are provided below.

P quantifies the accuracy of positive predictions by the model, defined as the ratio of correctly predicted positive samples to all samples predicted as positive. R measures the model’s ability to identify all actual positive samples, calculated as the ratio of correctly predicted positive samples to the total number of actual positive samples. The formulas for P and R are given in Equations 2, 3, Tang et al. (2023). Here, true positives (TP) are samples correctly predicted as positive, false positives (FP) are samples incorrectly predicted as positive, and false negatives (FN) are samples incorrectly predicted as negative when they are actually positive.


(2) $$P=\frac{TP}{TP+FP}$$

(3) $$R=\frac{TP}{TP+FN}$$

To evaluate model performance, a P-R curve is generated with R on the x-axis and P on the y-axis. The area under the P-R curve represents the Average Precision (AP), which measures the model’s detection accuracy for each category. A higher AP value indicates better detection performance. The mAP is the average of AP values across all categories and serves as a key metric for assessing overall model performance. mAP50 is the mAP value at an IoU threshold of 0.5 Yan et al. (2024), while mAP50–95 is the mAP value calculated across multiple IoU thresholds. Higher mAP values correspond to more accurate bounding box predictions. The formulas for AP and mAP are given in Equations 4, 5, where N represents the total number of categories.


(4) AP=∫01 P(r)dr

(5) $$mAP=\frac{\sum_{i=1}^{N}AP\left(i\right)}{N}$$

Additionally, we assess the lightweight characteristics of the model by considering its size, number of parameters, and FLOPs. Model size refers to the amount of memory the model occupies on the hardware. Parameters indicate the total count of trainable variables in the model, while FLOPs measure the computational workload required by the model. These metrics are crucial for evaluating model complexity and the computational resources needed Zhao et al. (2023).

### 2.6 Statistical analysis

Confidence intervals (CI) Shreffler and Huecker (2023) can be used to evaluate the reliability of the performance of the model and the significance of differences. The p-value is used to determine whether there are significant differences between models. P<0.05 indicates significant differences. In this study, IBM SPSS 20 Surendran et al. (2024) and a test set from the wild Ussurian Pear leaf dataset were used for the test. Compare the performance metrics of multiple models on the same test set to evaluate the performance of each model and verify whether the improved model in this paper has statistical significance.

## 3 Results and discussion

### 3.1 Experimental environment and parameter settings

This experiment is performed on a system running Ubuntu 20.04. Using Python 3.9.19 programming language. The development environment is CUDA 11.8. Use the PyTorch 2.0.0 deep learning framework. The GPU is NVIDIA GeForce RTX 3090. Equipped with 14 vCPU Intel (R) Xeon (R) Gold 6330 CPU @ 2.00GHz processor and 80GB memory.

The network training parameters are set as follows: the image input size is 640 × 640, the batch size is set to 16. The learning epoch is set to 200. Train the model using SGD as the optimizer and dynamically adjust the learning rate using the cosine annealing strategy. The initial learning rate is configured as 0.01, with a momentum factor of 0.937 and a weight decay coefficient of 0.0005. In this study, no pre-trained models are utilized; instead, all models are trained from scratch. To maintain experimental fairness, all models are trained under identical conditions. The ablation experiment uses the same hyperparameter settings, while the comparative experiments of different models use their own default hyperparameter settings.

### 3.2 Analysis of identification and classification results of wild Ussurian Pear leaves

**Figure 7:**
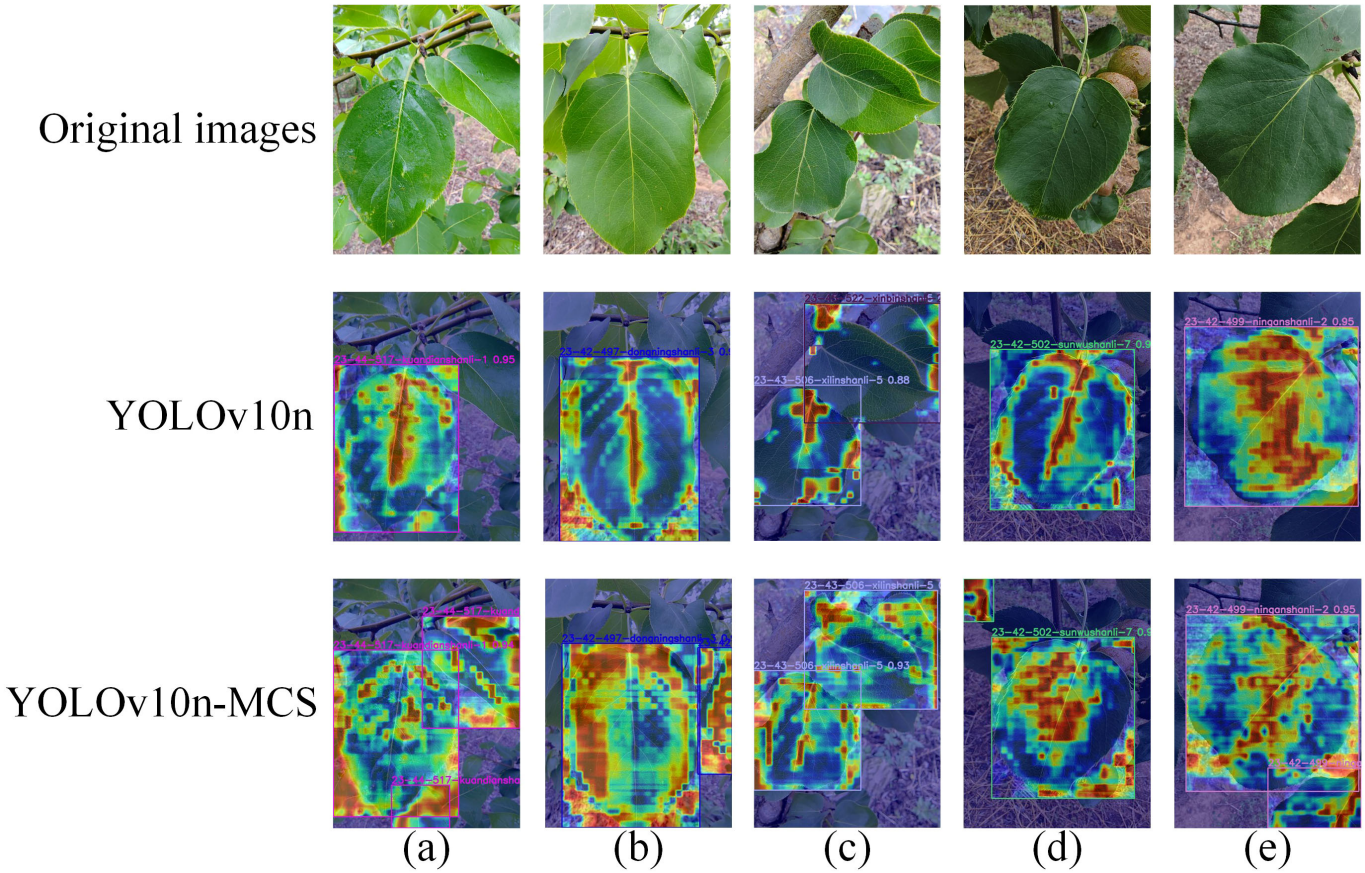
Grad-CAM heatmaps of some test set images: **(a)** *P. ussuriensis* ‘kuandian-1’, **(b)** *P. ussuriensis* ‘dongning-3’, **(c)** *P. ussuriensis* ‘xilin-5’, **(d)** *P. ussuriensis* ‘Sunwu-7’, **(e)** *P. ussuriensis* ‘ningan-2’.

The identification and classification of wild Ussurian Pear accessions in natural environments is crucial for the conservation of wild pear resources. To this end, the recognition and classification performance of the YOLOv10n-MCS model was tested using the self-made test set of wild Ussurian Pear leaves images dataset. Randomly select five images from the test set for heatmap visualization. Heatmap is a commonly used visual tool for displaying the importance of different regions in an image. The Gradient-weighted Class Activation Mapping (Grad-CAM) Selvaraju et al. (2017) method is employed to generate the model’s heatmap. Grad-CAM calculates the model classification weights and overlays them in the form of heatmap with the original image in equal proportions. To highlight important areas in the image. **Figure 7** shows the visual detection results of YOLOv10n and YOLOv10n-MCS on wild Ussurian Pear leaves. Among them, the areas of contribution to model detection are indicated in red and yellow, and the areas with less contribution are blue Wang et al. (2025b). By comparing the heatmap performance of YOLOv10n and YOLOv10n-MCS models in pear leaf classification and detection, significant differences in feature extraction can be found. The attention of the YOLOv10n model is highly focused on the central and lateral vein structures of the leaves, and the heatmap shows a clear linear distribution pattern, indicating that the model mainly relies on leaf vein morphological features for variety classification. The YOLOv10nMCS model demonstrates a more comprehensive feature capture capability, with its heatmap not only focusing on leaf vein structure, but also extensively covering the leaf surface, forming a more uniform activation distribution. This multi-scale feature extraction method enables it to simultaneously capture leaf morphology, texture, and overall contour features. YOLOv10n-MCS has achieved richer feature representation through its multi-channel strategy, which may have stronger discriminative ability and robustness in pear leaf variety classification tasks. This indicates that the method proposed in this study can improve the feature extraction ability of the model and achieve accurate and efficient recognition of wild Ussurian Pear leaves.

**Figure 8:**
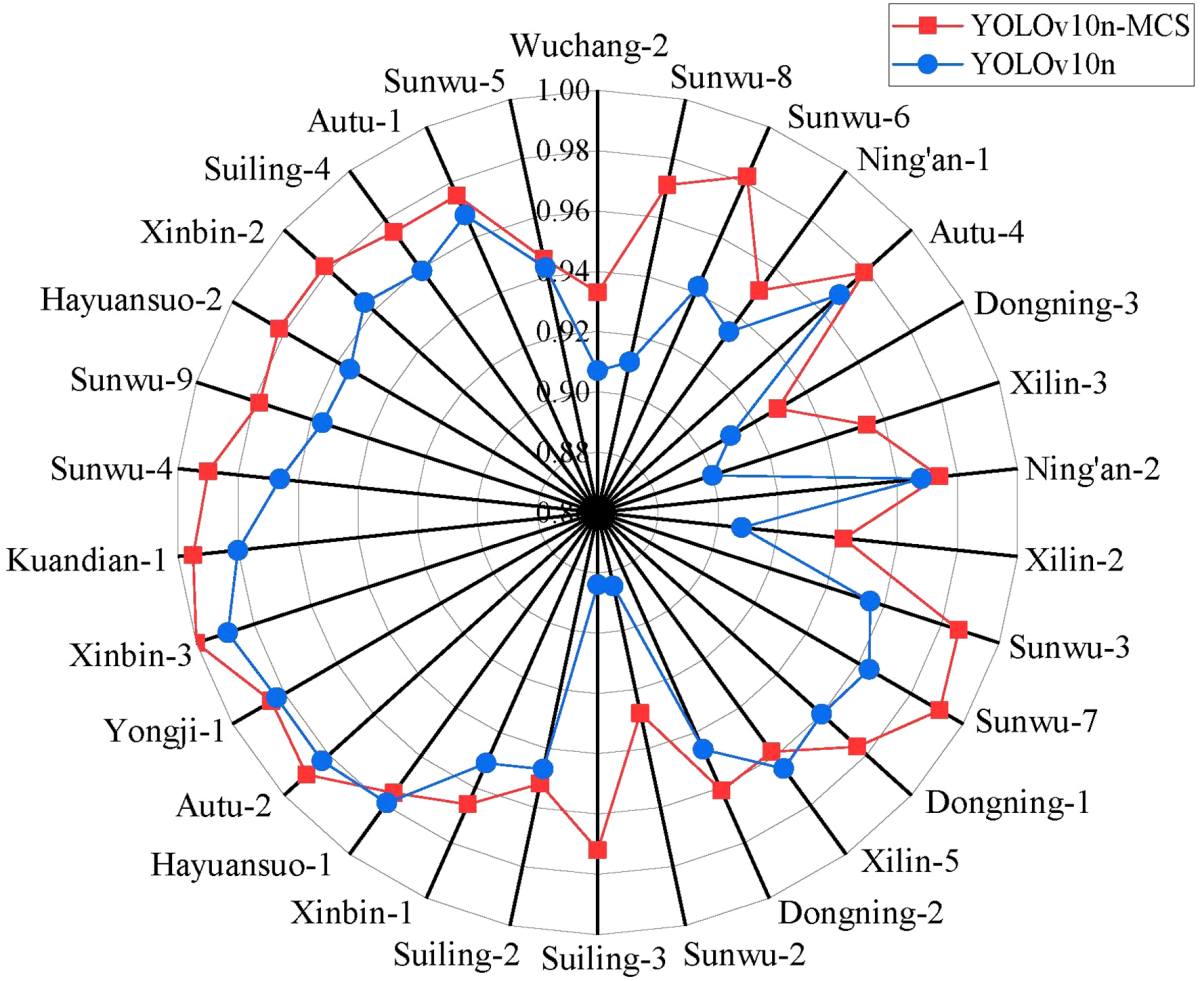
Precision results of 30 wild pear accessions.

In order to further evaluate the recognition and classification ability of YOLOv10n-MCS on wild Ussurian Pear leaves, **Table 2** shows the detection results of YOLOv10n and YOLOv10n-MCS on the recognition and classification of wild Ussurian Pear leaves. The results showed that YOLOv10n-MCS performed better in the task of identifying and classifying wild Ussurian Pear leaves. Compared with YOLOv10n, YOLOv10n-MCS has improved precision, recall, mAP50, and mAP50–95 by 2.9%, 2.3%, 1.5%, and 1.5%, respectively. In addition, the parameters, FLOPs, and model size of the model are reduced by 1.9M, 0.3G, and 0.4MB, respectively, and the model complexity is improved. Perform statistical analysis on precision, recall, and mAP separately. Among them, the precision of YOLOv10n is 94.8 (95% CI: 93.66 to 95.88)%, the recall is 91.2 (95% CI: 89.81 to 92.6)%, and the mAP is 97.3 (95% CI: 96.76 to 97.82)%. The precision of YOLOv10n-MCS is 97.7 (95% CI: 97.18 to 98.16)%, the recall is 93.5 (95% CI: 92.57 to 94.36)%, and the mAP is 98.8 (95% CI: 98.57 to 99.03)%. Compared with the original model, YOLOv10n-MCS showed better precision, recall, and mAP, and the difference is statistically significant (p<0.05). **Figure 8** shows the detection results of YOLOv10n and YOLOv10n-MCS on 30 wild Ussurian Pear accessions leaves. From the figure, it can be seen that YOLOv10n has slightly higher recognition precision for the two varieties ‘Xilin-5’ and ‘Xinbin-1’ than YOLOv10n-MCS. In addition, the recognition precision of the other 28 accessions, YOLOv10n-MCS is higher than YOLOv10n. Overall, YOLOv10n-MCS performs better in the task of identifying and classifying wild pear leaves, with a precision of over 92% for all accessions, meeting the demand for high recognition accuracy.

**Table 2:** The results of identifying and classifying wild Ussurian Pear leaves.

Model	Precision	Recall	mAP50	mAP50-95	Params	FLOPs	Model-size
	(%)	(%)	(%)	(%)	(M)	(G)	(MB)
YOLOv10n	94.8 [93.66-95.88]	91.2 [89.81-92.6]	97.3 [96.76-97.82]	93.2	2.71	8.5	5.8
YOLOv10n-MCS	97.7 [97.18-98.16]	93.5 [92.57-94.36]	98.8 [98.57-99.03]	94.7	2.52	8.2	5.4

Among them, the precision, recall, and mAP formats are: value [CI at 95% confidence level].

### 3.3 Ablation experiments

**Figure 9:**
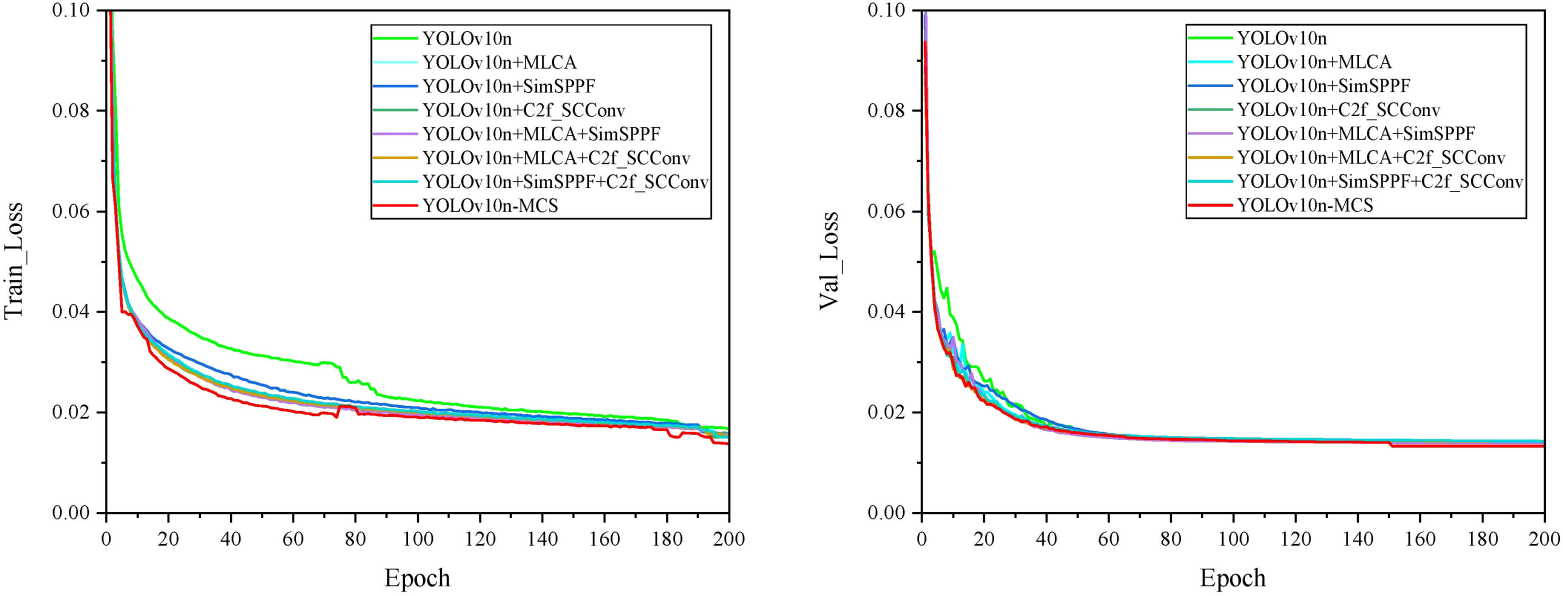
Loss curves of different models.

To assess the performance and practicality of the proposed YOLOv10n-MCS model in wild Ussurian Pear leaves identification and classification, ablation experiments were performed using the YOLOv10n model as the baseline. To maintain experimental consistency, the same dataset, environment, and parameter configurations were utilized throughout the experiments. The YOLOv10n-MCS model consists of three improved methods: MLCA, C2f SCConv, and SimSPPF. The ablation study investigated the effects of three enhancement techniques on model performance. **Figure 9** illustrates the loss curves for all models during training and validation. As shown in the figure, the loss function values exhibit a decreasing trend. With increasing epochs, both the training and validation loss curves gradually decline and stabilize, indicating that the models have effectively converged without underfitting or overfitting. The detailed results of the ablation experiments are presented in **Table 3**. Among them, “✓” represents the improvement methods that have been used.

**Table 3:** Comparison of ablation experiment results.

Methods	YOLOv10n	MLCA	SimSPPF	C2f SCConv	Precision	Recall	mAP50	mAP50-95	Params	FLOPs	Model-size
					(%)	(%)	(%)	(%)	(M)	(G)	(MB)
①	✓				94.8	91.2	97.3	93.2	2.71	8.5	5.8
②	✓	✓			97.1	93.6	98.2	94.1	2.71	8.7	5.8
③	✓		✓		96.6	93.8	98.2	94.2	2.71	8.5	5.8
④	✓			✓	96.9	92.5	97.9	93.7	2.52	7.9	5.4
⑤	✓	✓	✓		97.3	93.2	98.4	94.5	2.71	8.7	5.8
⑥	✓	✓		✓	96.5	93	98	93.8	2.52	8.2	5.4
⑦	✓		✓	✓	96.3	92.9	97.9	93.7	2.52	7.9	5.4
⑧	✓	✓	✓	✓	97.7	93.5	98.8	94.7	2.52	8.2	5.4

According to the experimental results in **Table 3**, it is found that all combinations improved the performance of the model compared to YOLOv10n. After introducing the MLCA module into the baseline network YOLOv10n (model ①), the precision, recall, mAP50, and mAP50–95 of model ② are improved by 2.3%, 2.4%, 0.9%, and 0.9%, respectively. The FLOPs have increased by 0.2G, and there is no significant change in the parameters compared to the original. MLCA divides the input feature map into multiple local blocks and preserves important spatial information within each block separately. Then calculate the global channel attention of the entire feature map separately. Combine local and global attention weights to create a comprehensive attention map that includes both local details and global contextual information. Weighting the original feature map with the comprehensive attention map highlights important features and suppresses irrelevant information. Finally, the enhanced feature map is input into the network, which improves the accuracy of detection and classification. The experimental results demonstrate that the MLCA module can effectively enhance the feature extraction capability of the model and achieve a better balance between detection performance, speed, and model parameter quantity. Therefore, introducing MLCA module can improve model performance without excessively increasing the parameters and FLOPs. After introducing the SimSPPF module separately, compared with the baseline model, model ③ improved its precision, recall, mAP50, and mAP50–95 by 1.8%, 2.6%, 0.9%, and 1%, respectively, without significant changes in the parameters and FLOPs. SimSPPF uses SimConv convolutional layers to extract features from the input feature map, accelerating the training process and improving the stability of the model, enhancing its expressive power. And through multiple max pooling and concatenation operations, the fusion of features at different scales is achieved. The simplified design improves computational efficiency and enhances the training speed of the model without increasing complexity. Similarly, after introducing the C2f SCConv module separately, the precision, recall, mAP50, and mAP50–95 of model ④ are improved by 2.1%, 1.3%, 0.6%, and 0.5%, respectively. At the same time, the parameters in the model decrease by 0.19M, the FLOPs decrease by 0.6G, and the model size decreases by 0.4MB. C2f SCConv introduces spatial and channel reconstruction modules in convolution operations. Learn the spatial correlation of feature maps through the spatial reconstruction module, and learn the channel correlation of feature maps through the channel reconstruction module. The use of segmentation transformation fusion strategy reduces redundancy and computational costs. By separating and reconstructing redundant features, spatial redundancy is suppressed and feature representation is enhanced. The experimental results indicate that the C2f SCConv module can reduce redundant features, decrease parameters, and lower FLOPs. It not only improve the model’s detection capabilities but also lowers its computational demands.

In addition, SimSPPF and C2f SCConv modules are introduced on the basis of MLCA module. It is found that when the SimSPPF module is introduced, the precision, recall, and mAP of model ⑤ do not significantly improve compared to model ②. The parameters, FLOPs, and model size remain unchanged, and the complexity of the model is not improved. When the C2f SCConv module is introduced, compared with model ②, the precision, recall, mAP50, and mAP50–95 of model ⑥ are reduced by 0.6%, 0.6%, 0.2%, and 0.3%, respectively. However, the parameters, FLOPs, and model size are reduced by 0.19M, 0.5G, and 0.4M, respectively, and the complexity of the model is reduced. Experiment ⑦ introduced the C2f SCConv module on top of the SimSPPF module in the baseline model. Compared with Experiment ③, its precision, recall, and mAP have decreased, but the parameters, FLOPs, and model size have decreased by 0.19M, 0.6G, and 0.4M, respectively. These results indicate that introducing a single module alone in the task of identifying and classifying wild pear leaves doesn’t effectively improve model performance, but may instead lead to a decrease in performance. Therefore, a holistic consideration of multiple factors is essential to enhance model performance.

Finally, the model introduced MLCA module, SimSPPF module, and C2f SCConv module simultaneously. The precision, recall, mAP50, and mAP50–95 of model ⑧ reached 97.7%, 93.5%, 98.8%, and 94.7%, respectively, which are improved by 2.9%, 2.3%, 1.5%, and 1.5% compared to model ①. At the same time, the parameters, FLOPs, and model size of the model are reduced by 0.19M, 0.3G, and 0.4MB, respectively. While improving the accuracy of the model in identifying wild Ussurian Pear leaves, the complexity of the model is reduced, thereby improving the detection performance of the model.

### 3.4 Comparison and analysis of different network models

To further validate the effectiveness of the YOLOv10n-MCS model, we compared it with eight mainstream object detection models, including two common models YOLOv7n and YOLOv8n, two other models in the YOLOv10 series YOLOv10s and YOLOv10m, VGG16 Simonyan (2014), ResNet50 He et al. (2016), RT-DETR Zhao et al. (2024), and the baseline model YOLOv10n. And analyze the experimental results. Train these eight models using the wild pear leaves dataset in the same experimental environment, with each model undergoing 200 iterations. Subsequently, the model is evaluated using a validation set and the results are compared with YOLOv10n-MCS.

**Figure 10:**
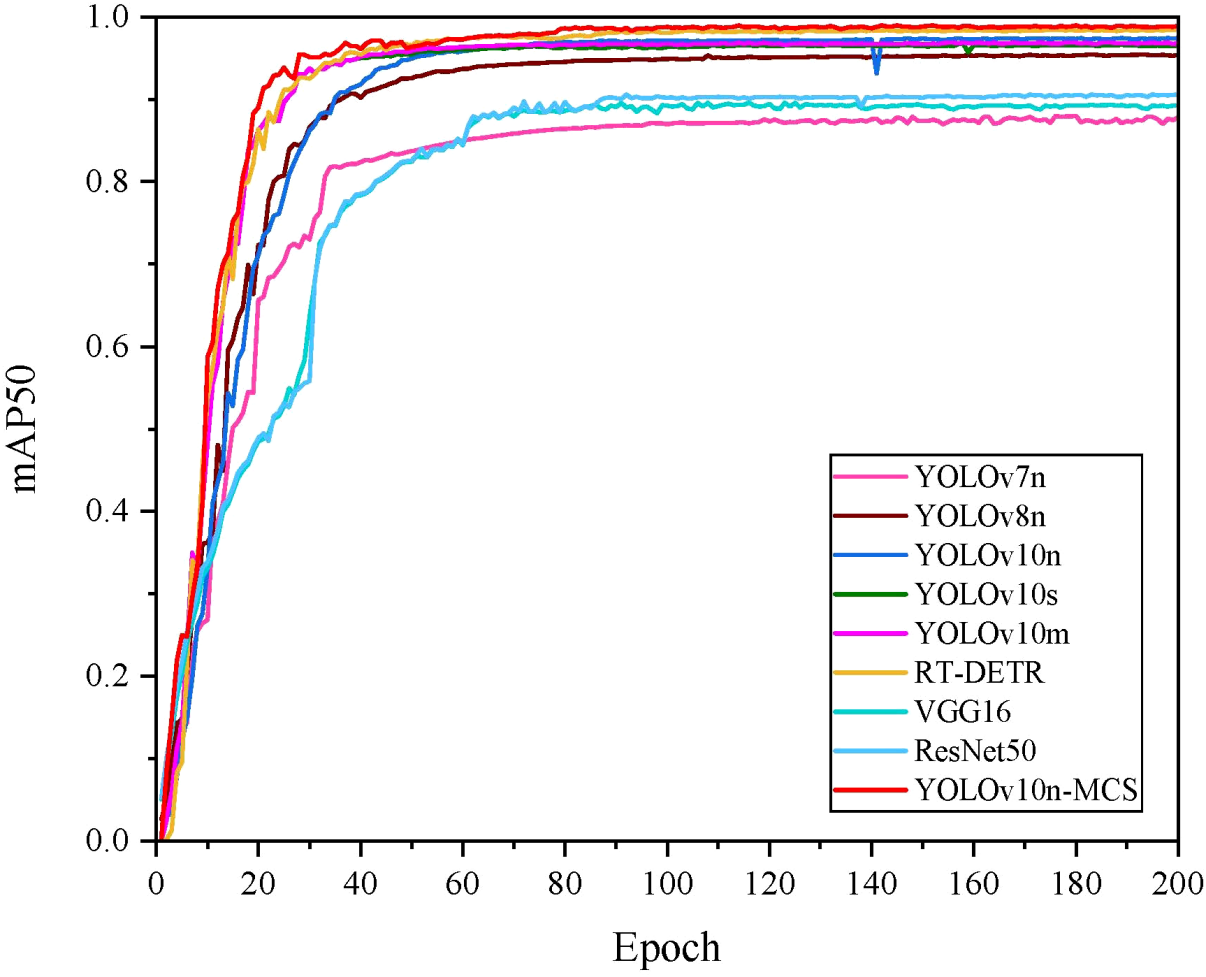
mAP50 curves of different detection models.

**Figure 11:**
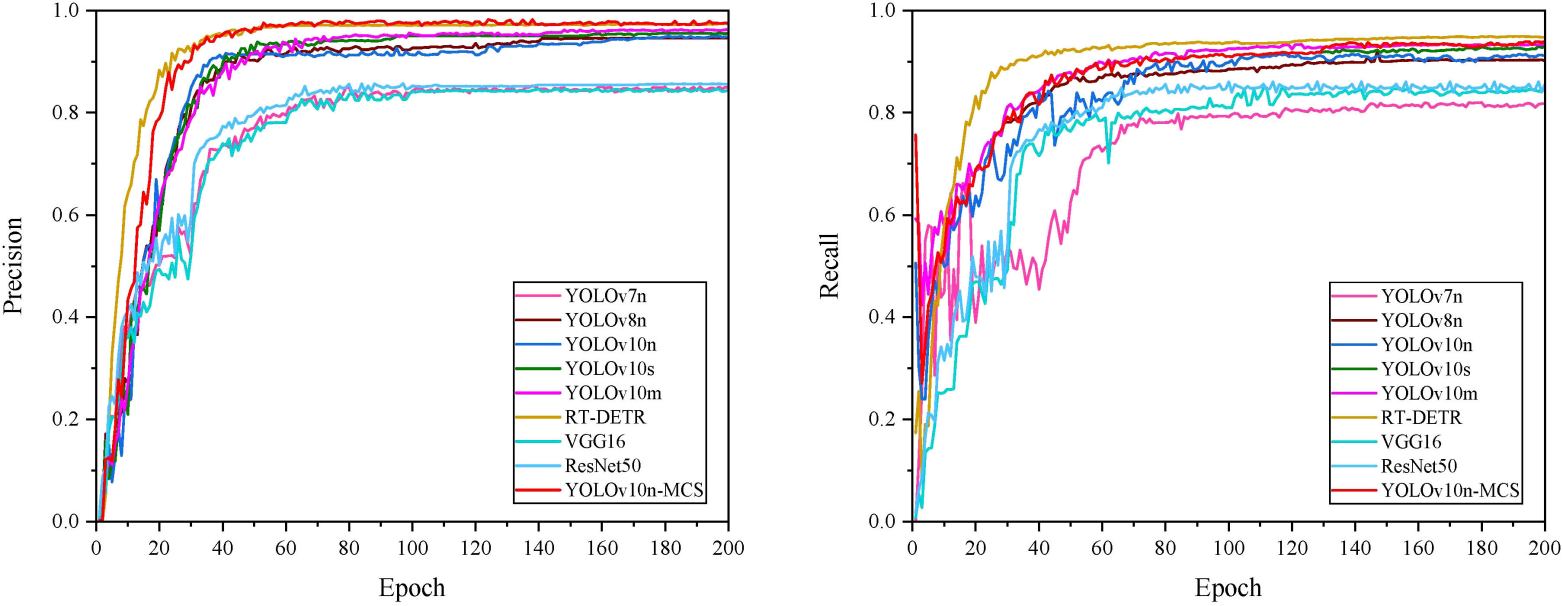
Precision and recall curves of different detection models.

**Figure 10** shows the mAP50 values for all models. It can be seen that the mAP50 value of YOLOv10nMCS is consistently better than other models. The precision and recall curves of all detection models are shown in **Figure 11**. **Table 4** displays detailed information on the recognition and classification results of all models. From the table, it can be seen that YOLOv10n-MCS achieves higher detection accuracy compared to other models. Its precision reached 97.7% and mAP50 reached 98.8%, higher than the other eight models. Its mAP50 values are 11%, 3.5%, 1.5%, 2.3%, 2%, 0.4%, 9.6%, and 8.4% higher than YOLOv7n, YOLOv8n, YOLOv10n, YOLOv10s, YOLOv10m, RT-DETR, VGG16, and ResNet50, respectively. YOLOv7n and YOLOv8n use traditional C3 modules with limited receptive fields and insufficient feature extraction capabilities. YOLOv10n-MCS uses large kernel deep convolution to expand the receptive field and enhance the detection capability of targets. And by introducing MLCA and SimSPPF modules, the feature extraction capability of the model has been effectively enhanced. The architecture design concept of VGG16 and ResNet50 adopts a classification oriented static architecture, while YOLOv10n-MCS solves the inherent defects of traditional classification models in object detection tasks through detection specific architecture design and dynamic feature fusion. Therefore, it is possible to more accurately identify and classify wild pear leaves.

**Table 4:** Comparison of detection performance of different models.

Model	Precision	Recall	mAP50	Params	FLOPs	Model-size
	(%)	(%)	(%)	(M)	(G)	(MB)
YOLOv7n	84.8 [83.44-86.24]	81.6 [80.51-82.82]	87.8 [86.25-89.14]	37.35	105.6	75.1
YOLOv8n	94.6 [93.43-95.71]	90.4 [89.51-91.36]	95.3 [94.91-95.75]	3.02	8.2	6.3
YOLOv10n	94.8 [93.66-95.88]	91.2 [89.81-92.6]	97.3 [96.75-97.81]	2.71	8.5	5.8
YOLOv10s	95.5 [94.8-96.26]	92.6 [91.73-93.51]	96.5 [96.21-96.93]	8.09	24.9	16.6
YOLOv10m	96.1 [95.31-96.91]	93 [91.99-93.97]	96.8 [96.54-96.99]	16.52	64.2	33.5
RT-DETR	97.3 [96.88-97.79]	94.9 [93.64-96.18]	98.4 [97.99-98.79]	32.87	108.1	66.3
VGG16	84.3 [82.94-85.74]	84.1 [83.16-85.08]	89.2 [88.30-90.18]	134.4	15.5	517.6
ResNet50	85.6 [84.28-86.97]	85.3 [84.55-86.14]	90.4 [89.83-90.93]	23.58	4.12	103.4
YOLOv10n-MCS	97.7 [97.18-98.16]	93.5 [92.57-94.36]	98.8 [98.57-99.02]	2.52	8.2	5.4

Among them, the precision, recall, and mAP formats are: value [CI at 95% confidence level].

Statistical analysis is conducted on the precision, recall, and mAP of YOLOv10n-MCS compared to 8 other models. Among them, its precision and mAP are significantly higher than YOLOv7n, YOLOv8n, YOLOv10n, YOLOv10s, YOLOv10m, VGG16, and ResNet50 (p<0.05). There is no significant difference comparad to RT-DETR (p=0.64 and 0.38, respectively). Its recall is significantly higher than YOLOv7n, YOLOv8n, YOLOv10n, VGG16, and ResNet50 (p<0.05). There is no significant difference between YOLOv10s and YOLOv10m (p=0.25 and 0.51, respectively). The recall of RT-DETR is significantly higher than that of YOLOv10n-MCS (p<0.05), but its parameters, FLOPs, and model size are 32.87M, 108.1G, and 66.3MB, respectively, which are too high compared to YOLOv10n-MCS. RT-DETR adopts an end-to-end detection architecture based on Transformer, which has computational redundancy and requires more computing resources and larger memory for training. High model complexity hinders its suitability for lightweight tasks. YOLOv10n-MCS uses depthwise separable convolution and channel compression techniques to reduce the model parameters and FLOPs. The FLOPs of YOLOv8n and YOLOv10n-MCS is the smallest, at 8.2G. However, YOLOv10n-MCS reduces redundancy and computational costs by introducing the C2f SCConv module. Compared to YOLOv8n, YOLOv10n-MCS has fewer parameters and lower model complexity. Its model size is 5.4MB, which is more lightweight compared to other models. Overall, the YOLOv10n-MCS model performs better. Therefore, it is more suitable for the task of identifying and classifying wild Ussurian Pear leaves.

## 4 Conclusion

Wild Ussurian Pear contains abundant genetic resources and is a good material for genetic improvement. Efficient and accurate identification and classification of wild Ussurian Pear accessions are the basis for resource collection, preservation, research and utilization. At present, there are no reports on the classification of wild pear germplasm resource by identifying leaves. Therefore, this article collected wild Ussurian Pear leaves images in the natural background and constructed a dataset of leaves images covering 30 accessions. And using YOLOv10n as the baseline model, a lightweight model called YOLOv10n-MCS was proposed for the recognition and classification of wild Ussurian Pear leaves in complex scenes. We have introduced the MLCA module based on YOLOv10n to enhance the feature extraction capability of the model. Use SimSPPF module instead of SPPF in the baseline model to improve the detection efficiency of the model. C2f SCConv module was designed to replace C2f in the original network backbone, reducing the computational redundancy of the model. We used a dataset of wild Ussurian Pear leaves images to validate the performance of the improved model. The experimental results showed that YOLOv10n-MCS achieved recognition precision, recall, mAP50, and mAP50–95 of 97.7%, 93.5%, 98.8%, and 94.7% for 30 accessions of wild Ussurian Pear leaves, respectively. The precision of 18 wild pear accessions can reach over 97%, while the precision of the other 12 accessions remains between 92% and 97%. Among them, the recognition precision of *P. ussuriensis* ‘Xinbin-1’ can reach 100%, meeting the accuracy requirements for wild Ussurian Pear leaves recognition and classification tasks. Statistical analysis was conducted on YOLOv10n and YOLOv10n-MCS, and the results showed that YOLOv10n-MCS exhibited statistically significant improvements in precision (2.9% improvement, p<0.05), recall (2.3% improvement, p<0.05), and mAP (1.5% improvement, p<0.05). Further validated the effectiveness of the improvement, particularly in terms of precision and recall.

Comparing the performance of the model with 8 mainstream models, the results show that YOLOv10nMCS has advantages in recognition precision, recall, and model size. It can improve detection performance while reducing parameter and FLOPs. This model can quickly and accurately identify wild Ussurian Pear leaves, outperforming baseline model and other mainstream models in automatic recognition and classification tasks of wild pear leaves.

This study demonstrates the feasibility of using object detection algorithms to identify wild Ussurian Pear leaves for accessions classification. This method can quickly identify wild Ussurian Pear germplasm resource while ensuring accuracy, reducing labor costs. The model proposed in this article effectively meets the requirements of accuracy and real-time performance, which helps to automate the identification of wild Ussurian Pear accessions and provides technical support and reference for the protection, utilization, classification research of wild pear germplasm resource. However, due to the limited sample size in this experiment and significant differences between different populations, its widespread use still requires continuous optimization of performance according to specific requirements and adaptation to new challenges. Nevertheless, the results of this study still provide a good template for achieving this goal.

## Data Availability

The raw data supporting the conclusions of this article will be made available by the authors, without undue reservation.
