# Supplementary figures and images for: Identification of leaves of wild Ussurian Pear (Pyrus ussuriensis) based on YOLOv10n-MCS
Source: Front Plant Sci. 2025 Jul 3;16:1588626. doi: 10.3389/fpls.2025.1588626 (PMC12267203; doi:10.3389/fpls.2025.1588626)

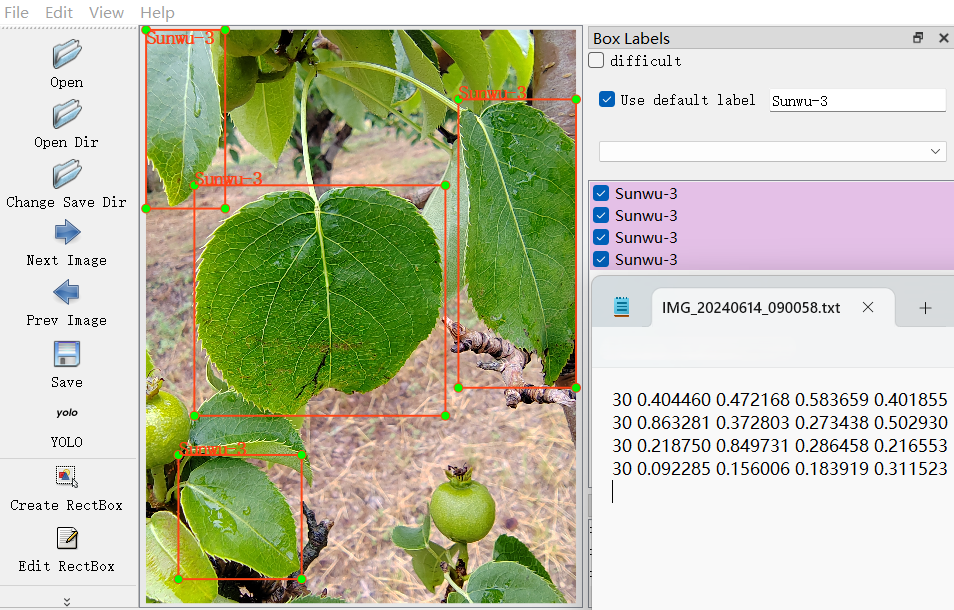

Supplement: Supplementary file 1 [file Image1.jpeg]
